# Associations between intrinsic capacity, plasma p-tau181 and cognitive function over a 5-year follow-up among community-dwelling older adults: a secondary analysis of the MAPT Study

**DOI:** 10.1016/j.tjfa.2025.100064

**Published:** 2025-07-01

**Authors:** Kelly Virecoulon Giudici, Philipe de Souto Barreto, Christelle Cantet, Henrik Zetterberg, Kaj Blennow, Bruno Vellas, for the MAPT/DSA group

**Affiliations:** a IHU HealthAge, Gerontopole of Toulouse, Institute of Ageing, Toulouse University Hospital (CHU Toulouse), Toulouse, France; b CERPOP UMR1295, University of Toulouse III, Inserm UPS, Toulouse, France; c Department of Psychiatry and Neurochemistry, Institute of Neuroscience and Physiology, the Sahlgrenska Academy at University of Gothenburg, Mölndal, Sweden; d Clinical Neurochemistry Laboratory, Sahlgrenska University Hospital, Mölndal, Sweden; e Department of Neurodegenerative Disease, UCL Institute of Neurology, Queen Square, London, United Kingdom; f UK Dementia Research Institute at UCL, London, United Kingdom; g Hong Kong Center for Neurodegenerative Diseases, Clear Water Bay, Hong Kong, PR China; h Wisconsin Alzheimer’s Disease Research Center, University of Wisconsin School of Medicine and Public Health, University of Wisconsin-Madison, Madison, WI, USA

**Keywords:** P-tau181, Intrinsic capacity, Cognitive function, Aging, Older adults

## Abstract

**Background:**

Intrinsic capacity (IC) is a recent key concept proposed by the World Health Organization (WHO) based on aspects of functional ability (both physical and mental) rather than the presence or absence of diseases, with a potential to predict several health outcomes.

**Objective:**

To explore associations between IC and cognitive function (prospectively), and between IC and plasma p-tau181 (cross-sectionally and prospectively) among community-dwelling older adults.

**Methods:**

Observational study with 491 subjects ≥70 years (67.4 % female, mean 75.3 years, SD=4.4), participants from the Multidomain Alzheimer Preventive Trial (MAPT). IC domains (locomotion, cognition, psychological, vitality) were combined into a 0–100 score. Alternative classification was based on the number of domains’ abnormalities. Plasma p-tau181 was measured at baseline and 36 months of follow-up. A composite cognitive score (CCS) based on four tests was determined at baseline, 6, 12, 24, 36, 48 and 60 months.

**Results:**

Inverse cross-sectional associations were observed between baseline IC score and p-tau181 (unadjusted model: β=-0.08, 95 %CI -0.13 to -0.03; *p* = 0.0025). A significant mean difference in p-tau181 3-year changes was observed between participants with low and normal IC (based on IC score) (adjusted model: 1.71, 95 %CI 0.01 to 3.40; *p* = 0.0483). Prospective 5-year associations between IC and CCS were only observed in unadjusted analysis according to the alternative IC classification (-0.21, 95 %CI -0.38 to -0.04; *p* = 0.0156).

**Conclusion:**

IC was associated with plasma p-tau181 and cognitive function, but findings varied according to the method of IC classification. Further research may help settle the role of IC as a predictor of neurodegenerative diseases such as AD. In this regard, multidomain interventions have potential to protect IC over the aging process and prevent cognitive impairment, and should also be encouraged.

**Abbreviations:** AD, Alzheimer’s disease; BMI, body mass index; CCS, composite cognitive score; CI, confidence interval; CNT, Category Naming Test; Digit Symbol Substitution Test; GDS, Geriatric Depression Scale; IC, intrinsic capacity; MAPT, Multidomain Alzheimer Preventive Trial; MCI, mild cognitive impairment; MMSE, Mini-Mental State Examination; p-tau 181, phosphorylated-tau 181; PUFA, polyunsaturated fatty acid; Q, quartile; SAS, Statistical Analysis Software; SPPB, Short Physical Performance Battery; WHO, World Health Organization.

## 1 Introduction

Since its conceptualization by the World Health Organization (WHO) in 2015 [1], intrinsic capacity (IC) has been increasingly recognized as an important measure in clinical practice and research related to aging and public health of older adults. Based on aspects of functional ability (both physical and mental) rather than the presence or absence of disease [1], IC is now the target of multiple research in the sake of finding effective interventions to promote healthy aging and to improve quality of life [2]. Its five domains (locomotion, cognition, psychological, sensory – composed by hearing and vision – and vitality) reflects the biological reserves of the individual, what can be translated into biological age [3,4].

Following the extension of life expectancy, the prevalence of neurodegenerative diseases related to cognitive decline are widely increasing worldwide [5,6]. In the meanwhile, technological advances are enabling research on the topic to reduce costs and complexity by exploring neurodegenerative biomarkers in blood. In this sense, phosphorylated-tau 181 (p-tau181) assessed in plasma was the first p-tau specie to demonstrate good accuracy for discriminating subjects with AD versus controls [[7], [8], [9], [10]] and has been widely used by researchers in recent years [11,12]. Together with clinical assessments, it might help understanding the biological processes leading to cognitive impairment and the factors related to the speed of its advance over time.

Although increasing evidence shows IC to be a predictor of disability, falls, hospitalization, frailty and mortality in middle-aged and older adults [[13], [14], [15]], very limited research exploring the associations between IC and outcomes related to neurodegeneration is available so far. Initial evidence points to higher IC acting as a protective factor against the incidence of Alzheimer’s disease (AD) and vascular dementia [16]. If this relationship is supported by further studies, strategies protecting IC in older adults could possibly help fighting the burden of neurodegenerative diseases. The present study aimed to investigate cross-sectional and prospective associations between IC (defined with two different classifications), plasma p-tau181 and cognitive function (assessed by a composite score based on clinical tests) among community-dwelling older adults at risk of cognitive decline.

## 2 Methods

This prospective observational study was performed using data from the Multidomain Alzheimer Preventive Trial (MAPT), a study created to investigate the efficacy of a 3-year omega-3 polyunsaturated fatty acid (PUFA) supplementation and a multidomain intervention based on nutritional counseling, physical activity and cognitive training in preventing cognitive decline among community-dwelling older adults. MAPT interventions, either combined or alone, had no significant effects on cognitive decline in the studied population, and participants were additionally followed for another two years (observational phase). Details are described elsewhere [17,18].

### 2.1 Study population

MAPT participants were men and women free of dementia aged ≥70 years with spontaneous memory complaints, limitation in executing at least one instrumental daily activity, or slow gait speed (≤0.8m/s measured by a 4-meter usual walking test), living in France. Further information about participants’ selection is available elsewhere [17]. Data was collected in 13 health centers from May 2008 to February 2011 (recruitment), until April 2016 (end of follow-up).

### 2.2 Ethical considerations

Subjects who agreed to join the study signed an informed consent. The study protocol (NCT00672685, available at www.clinicaltrials.gov) was authorized by the French Health Authority and approved by the Advisory Committee for the Protection of Persons participating in Biomedical Research of the Toulouse University Hospital.

### 2.3 Intrinsic capacity assessment

IC domains were assessed by trained personnel, and for this study only baseline IC was considered. Because data for the sensory domain of IC was not available for MAPT participants, four domains were evaluated: locomotion, cognition, psychological, and vitality. IC domains were combined into a 0-100 point scale (in which higher scores indicate higher/better IC), as previously described by Lu et al. [19]. Locomotion was assessed by the Short Physical Performance Battery (SPPB), a 12-point scale [20]. Cognitive function was assessed by the Mini Mental State Examination (MMSE), a 30-point instrument [21]. The psychological domain was evaluated by the Geriatric Depression Scale (GDS), a 15-item instrument for identifying depressive symptoms in older adults with scores ranging from 0 to 15 (with higher scores meaning worse) [22]. Vitality was based on the measure of hand grip strength (determined in kg with a handheld dynamometer in the dominant hand) [23]. Each test was then converted to a 0–100-point scale, with different maximum values applied for each sex for hand grip strength. Then, points were summed and divided by 4 (with the rescaled GDS score weighted as −1).

In the present study, IC was additionally assessed according to the number of abnormalities on its domains. Abnormality in locomotion was defined as SPPB score < 10 [24]. For cognitive function, abnormality was considered if MMSE score < 26 [25]. Abnormal psychological domain was defined as GDS score ≥5 [22]. Vitality, in turn, was considered as abnormal if handgrip strength ≤ first quartile (Q1) (different values were applied for each sex). The number of abnormalities was then summed to form an IC score of 0-4 points.

### 2.4 Cognitive function assessment (as an outcome)

Cognitive function analyzed as an outcome was assessed by a composite cognitive score (CCS) based on the sum of Z-scores from four cognitive tests divided by 4: free and total recall of the Free and Cued Selective Reminding test, the ten orientation items of the MMSE, the Digit Symbol Substitution Test and the Category Naming Test [18,26]. This measure was assessed at baseline, 36 and 60 months of follow-up.

### 2.5 Plasma p-tau181 assessment

Plasma p-tau181 concentrations were assessed in a subset of MAPT participants (*n* = 558 out of 1679) by using stored blood samples (frozen at −80 °C in EDTA tubes) taken at baseline and after 3 years of follow-up. A Single molecule array (Simoa) method based on the AT270 (specific for the threonine-181 phosphorylation site), and Tau12 (N-terminal epitope 6–18 on human tau protein) anti-tau antibodies was performed, as previously detailed elsewhere [8], by using an HD-X Analyzer (Quanterix, Billerica, MA, USA) in a single batch analysis. P-tau181 values more than 3 standard deviations (SD) above the mean or less than 1 pg/mL were considered as outliers and excluded from this analysis. Excluding outliers resulted in 527 subjects. From them, 491 had information to build the IC score and were included in the present study.

### 2.6 Confounders

Analyses were adjusted by sex, age (years), education (no diploma or primary school certificate, secondary education, high school diploma or university level), body mass index (in kg/m^2^), number of comorbidities (considering diabetes, hypertension, hypercholesterolemia, cardiovascular disease, active cancer, asthma and chronic obstructive pulmonary disease) and their interaction with time.

### 2.7 Statistical analysis

Participants of the study were characterized by using descriptive statistics. Linear regression analysis was used to compare explore the associations between continuous IC score and p-tau181 levels at baseline. Sensitivity analysis with IC score in quartiles was also tested. Linear mixed effects models (with a random effect at the subject level and at the center level, for the CCS) were performed to explore the variation in plasma p-tau181 and in the CCS (dependent variables) according to IC groups (Q1 vs. Q2 to Q4 based on the 0–100 points score) over time (from baseline to 36 months for plasma p-tau181 and from baseline to 36 and 60 months of follow-up for the CCS). First, unadjusted linear mixed models were performed with the following fixed effects: IC group, time, and their interaction. Then, adjusted linear mixed models were performed, taking into account the potential confounders mentioned above and their interaction with time (model 1: only considering age, sex and education; model 2: considering all variables). Sensitivity analysis with the alternative IC classification, based on the number of IC domains’ abnormalities (≥1 vs 0), were performed similarly. For prospective analyses, only participants from the control group of the MAPT Study were included (those taking placebo and not performing the multidomain intervention). Since MMSE (as a measure of the cognitive domain) was used to build IC score, in analyses testing the associations between IC at baseline and cognitive function over time, two versions of IC score were considered: one including the MMSE and another excluding it (thus considering only three IC domains). Analyses were performed with the Statistical Analysis Software (SAS) version 9.4 (Cary, NC, USA), with a significance level set as 5 %.

## 3 Results

### 3.1 Characterization of the study sample

The studied sample had mean age of 75.3 years (SD=4.4), was composed by 67.4 % women and presented mean plasma p-tau 181 concentrations of 10.0 pg/mL (SD=4.5). Mean IC score was 78.5 (SD=7.6), and 54.6 % of the sample presented at least one abnormality in IC domains’. Additional participants’ characteristics at baseline are presented in Table 1.

**Table 1:** Characteristics of the studied sample.

	Total *n* = 491					
	n		%	mean	SD	
						
Sex (female)	331		67.4	–	–	
Age (years)	491		–	75.3	4.4	
Education						
No diploma or primary school certificate	113		23.3	–	–	
Secondary education	165		34.0	–	–	
High school diploma	68		14.0	–	–	
University level	140		28.8	–	–	
Body mass index (kg/m²)	490		–	26.3	4.0	
Number of comorbidities						
0	142		28.9	–	–	
1	195		39.7	–	–	
≥2	154		31.4	–	–	
						
Intrinsic capacity domains and status						
Intrinsic capacity score (0–100)	491		–	78.5	7.6	
*Cognition*						
MMSE score (0–30)	491		–	28.1	1.6	
*Psychological*						
GDS score (0–15)	491		–	3.1	2.6	
*Locomotion*						
SPPB score (0–12)	491		–	10.7	1.6	
*Vitality*						
Hand grip strength (kg)	491		–	26.6	8.9	
Intrinsic capacity status according to abnormalities in domains	491					
Low IC (≥ 1 abnormality)	268		54.6	–	–	
Normal IC (0 abnormality)	223		45.4	-	–	
						
**Outcomes**						
Plasma p-tau181 (pg/mL)	491		–	10.0	4.5	
Composite cognitive score	491		–	0.0	0.7	
Free and total recall of the FCSR score	491			73.5	9.1	
10-orientation items of the MMSE score	491			9.8	0.4	
Digit Symbol Substitution Test score	491			38.0	10.3	
Category Naming Test score	491			26.2	7.2	

FCSR, Free and Cued Selective Reminding test; GDS, Geriatric Depression Scale; IC, intrinsic capacity; MMSE, Mini-Mental State Examination; p-tau 181, phosphorylated-tau 181; SPPB, Short Physical Performance Battery; SD, standard deviation.

### 3.2 Cross-sectional associations between baseline IC and plasma p-tau181

We observed an inverse association between baseline IC score and baseline plasma p-tau181 (β=−0.08, 95 % CI −0.13 to −0.03; *p* = 0.003), but significance did not persist after adjusting for potential confounders (model 1: β=−0.04, 95 % CI −0.10 to 0.01; *p* = 0.120; model 2: β=−0.05, 95 % CI −0.10 to 0.01; *p* = 0.096) (Table 2). No cross-sectional associations were found when considering the alternative IC classifications based on the presence of domains’ abnormalities (Table 2).

**Table 2:** Cross-sectional associations between baseline intrinsic capacity and plasma p-tau181 among community-dwelling older adults.

			Baseline plasma p-tau181		
		n	β	95 % CI	p-value
Intrinsic capacity score (0–100)					
Unadjusted		491	−0.08	−0.13 to-0.03	**0.003**
Adjusted (model 1)**		486	−0.04	−0.10 to 0.01	0.120
Adjusted (model 2)***		485	−0.05	−0.10 to 0.01	0.096
					
Intrinsic capacity status* (0–4, ≥1vs 0)					
Unadjusted		511	0.57	−0.22 to 1.36	0.150
Adjusted (model 1)**		504	−0.06	−0.87 to 0.75	0.886
Adjusted (model 2)***		504	−0.01	−0.83 to 0.80	0.971

CI, confidence interval; IC, intrinsic capacity; *based on the number of abnormalities in IC domains at baseline: abnormality in cognitive function if MMSE <26; abnormality in locomotion if SPPB <10; abnormality in vitality if grip strength ≤Q1 (different values were applied for each sex); abnormality in psychological domain if GDS ≥5; In spite of *n* = 491 subjects with information in all the 4 domains used to define IC status, 20 additional subjects with p-tau181 assessment had information on at least 1 IC domain and presented abnormality on it, reaching a sample of 511 for this specific analysis. **Model 1: adjusted by sex, age (years), education (no diploma or primary school certificate, secondary education, high school diploma or university level) and their interaction with time. ***Model 2: adjusted by sex, age (years), education (no diploma or primary school certificate, secondary education, high school diploma or university level), BMI (kg/m^2^), and number of comorbidities (considering diabetes, hypertension, hypercholesterolemia, cardiovascular disease, active cancer, asthma or chronic obstructive pulmonary disease).

In sensitivity analysis with baseline IC score in quartiles, higher plasma p-tau was observed among participants in the forth quartile of IC score, compared to those in Q1 (β=−1.67, 95 % CI −2.80 to −0.55; *p* = 0.004), while no associations were observed when Q2 or Q3 were compared to Q1. This finding did not remain significant in the adjusted models though (model 1: β=−1.06, 95 % CI −2.21 to 0.09; *p* = 0.072; model 2: β=−1.11, 95 % CI −2.27 to 0.04; *p* = 0.058) (Supplementary Table 1).

### 3.3 Prospective associations between IC and 3-year plasma p-tau181

After 3 years, plasma p-tau changes remained stable among participants with low IC (Q1 of the IC score), but slightly declined among those with normal IC (Q2 to Q4 of IC score). In spite of this change did not reach statistical significance (*p* = 0.095), when the changes over time of these two groups were compared, a significant mean difference between-groups was observed in the full model adjusted for potential confounders (model 2: 1.71, 95 %CI 0.01 to 3.40; *p* = 0.048) (Table 3).

**Table 3:** Mixed-effect linear regression analysis for variation in plasma p-tau181 over a 3-year follow-up according to baseline intrinsic capacity among community-dwelling older adults (participants of the MAPT Study in the placebo group).

	Change in plasma p-tau181 (pg/mL) from baseline to 3 years		Group difference in plasma p-tau181 between low IC and normal IC (ref.) over 3 years		
	Within-group mean difference (95 % CI); n*		Between-group mean difference (95 % CI); P-value		
	Low IC	Normal IC	Unadjusted	Adj. (model 1)	Adj. (model 2)
**Baseline intrinsic capacity score (0–100)**	**Q1 (≤76.96)**	**Q2 to Q4**			
Baseline to 3 years	0.09 (−1.22, 1.40); *p* = 0.890; *n* = 33 to 32	−0.67 (−1.45, 0.12); *p* = 0.095; *n* = 92 to 91	0.76 (−0.77, 2.29); 0.328	1.65 (−0.04, 3.33); 0.055	1.71 (0.01, 3.40); **0.048**
					
Baseline intrinsic capacity status** (0–4)	**≥1 abnormalities**	**0 abnormalities**			
Baseline to 3 years	−1.01 (−1.90, −0.13); ***p*=0.026**; *n* = 73 to 71	−0.01 (−1.00, 0.99); *p* = 0.991; *n* = 57 to 57	−1.01 (−2.34, 0.32); 0.137	−0.56 (−2.08, 0.95); 0.463	−0.63 (−2.14, 0.87); 0.405

CI, confidence interval; IC, intrinsic capacity; *n=baseline to 3 years; **based on the number of abnormalities in IC domains at baseline: abnormality in cognitive function if MMSE <26; abnormality in locomotion if SPPB <10; abnormality in vitality if grip strength ≤Q1 (different values were applied for each sex); abnormality in psychological domain if GDS ≥5; Model 1: adjusted by sex, age (years), education (no diploma or primary school certificate, secondary education, high school diploma or university level) and their interaction with time. Model 2: adjusted by sex, age (years), education (no diploma or primary school certificate, secondary education, high school diploma or university level), BMI (kg/m^2^), number of comorbidities (considering diabetes, hypertension, hypercholesterolemia, cardiovascular disease, active cancer, asthma or chronic obstructive pulmonary disease) and their interaction with time.

In sensitivity analysis considering the alternative classification of IC based on the number of abnormalities, a significant decline in plasma p-tau181 was observed among participants with low IC (−1.01, 95 % CI −1.90 to −0.13; *p* = 0.026), but not in the normal IC group. However, no differences were observed for 3-year changes in plasma p-tau181 between groups (adjusted model 1: −0.56, 95 % CI −2.08 to 0.95; *p* = 0.463; model 2: −0.63, 95 % CI −2.14 to 0.87; *p* = 0.405) (Table 3).

### 3.4 Prospective associations between IC and cognitive function

Cognitive function significantly declined over time (after 3 years and 5 years), independently of IC classification (low or normal) based on the 0–100 points score. However, no between-group mean differences were observed, for both IC constructions (including the MMSE or not) (Table 4). When evaluating the IC score in separate quartiles, significant declines in CCS over time were observed only among participants in Q1 and Q2 of IC score (both at 3 and 5 years, and independently of including MMSE or not; at 5 years in the version excluding the MMSE, significant declines were also observed among participants in Q3), but no between-groups mean differences were observed when comparing Q2, Q3 and Q4 to Q1 (Supplementary Table 2).

**Table 4:** Mixed-effect linear regression analysis for variation in composite cognitive score over a 5-year follow-up according to baseline intrinsic capacity among community-dwelling older adults (participants of the MAPT Study in the placebo group).

	Change in composite cognitive score** from baseline to 3 or 5 years		Group difference in composite cognitive score** between low IC and normal IC (ref.) over time		
	Within-group mean difference (95 % CI); n*		Between-group mean difference (95 % CI); P-value		
	Low IC	Normal IC	Unadjusted	Adj. (model 1)	Adj. (model 2)
**Intrinsic capacity score (0–100, MMSE included)**	**Q1 (≤75.06)**	**Q2 to Q4**			
Baseline to 3 years	−0.16 (−0.30, −0.01); ***p*** **=** **0.036**; *n* = 93 to 67	−0.10 (−0.19, −0.02); ***p*** **=** **0.015**; *n* = 263 to 220	−0.05 (−0.22, 0.11); 0.533	0.05 (−0.12, 0.22); 0.576	0.04 (−0.13, 0.22); 0.609
Baseline to 5 years	−0.30 (−0.49, −0.11); ***p*** **=** **0.002**; *n* = 93 to 39	−0.19 (−0.29, −0.09); ***p*** **=** **0.0002**; *n* = 263 to 172	−0.11 (−0.32, 0.11); 0.331	−0.02 (−0.23, 0.20); 0.880	−0.03 (−0.25, 0.19); 0.805
**Intrinsic capacity score (0–100, MMSE excluded)**	**Q1 (≤68.65)**	**Q2 to Q4**			
Baseline to 3 years	−0.16 (−0.31, −0.01); ***p*** **=** **0.033**; *n* = 89 to 63	−0.10 (−0.18, −0.02); ***p*** **=** **0.015**; *n* = 267 to 224	−0.06 (−0.23, 0.11); 0.485	0.04 (−0.13, 0.22); 0.640	0.04 (−0.14, 0.21); 0.683
Baseline to 5 years	−0.31 (−0.50, −0.12); ***p*** **=** **0.002**; *n* = 89 to 40	−0.19 (−0.29, −0.09); ***p*** **=** **0.0002**; *n* = 267 to 171	−0.11 (−0.33, 0.10); 0.297	−0.02 (−0.24, 0.19); 0.847	−0.03 (−0.25, 0.18); 0.774
Intrinsic capacity status*** (0–4, MMSE included)	**≥1 abnormalities**	**0 abnormalities**			
Baseline to 3 years	−0.18 (−0.27, −0.09); ***p*** **=** **0.0001**; *n* = 219 to 167	−0.01 (−0.12, 0.10); *p* = 0.872; *n* = 149 to 132	−0.17 (−0.31, −0.03); **0.018**	−0.08 (−0.23, 0.07); 0.312	−0.08 (−0.22, 0.07); 0.317
Baseline to 5 years	−0.30 (−0.41, −0.19); ***p*** **<** **0.0001**; *n* = 219 to 113	−0.09 (−0.22, 0.04); *p* = 0.169; *n* = 149 to 109	−0.21 (−0.38, −0.04); **0.016**	−0.09 (−0.27, 0.08); 0.293	−0.09 (−0.27, 0.09); 0.316
Intrinsic capacity status*** (0–3, MMSE excluded)	**≥1 abnormalities**	**0 abnormalities**			
Baseline to 3 years	−0.15 (−0.24, −0.05); ***p*** **=** **0.002**; *n* = 206 to 158	−0.07 (−0.17, 0.04); *p* = 0.199; *n* = 161 to 140	−0.08 (−0.22, 0.06); 0.2546	0.01 (−0.13, 0.16); 0.8662	0.01 (−0.13, 0.16); 0.860
Baseline to 5 years	−0.29 (−0.41, −0.17); ***p*** **<** **0.0001**; *n* = 206 to 107	−0.13 (−0.25, −0.00); ***p*** **=** **0.044**; *n* = 161 to 114	−0.16 (−0.33, 0.01); 0.0635	−0.04 (−0.21, 0.14); 0.6715	−0.04 (−0.22, 0.13); 0.645

CI, confidence interval; IC, intrinsic capacity; MMSE, Mini Mental State Examination; Q, quartile; *n=baseline to 3 years or 5 years; **based on the sum of Z-scores from four cognitive tests divided by 4: free and total recall of the Free and Cued Selective Reminding test, the ten orientation items of the Mini-Mental State Examination, Digit Symbol Substitution Test and Category Naming Test; ***based on the number of abnormalities in IC domains at baseline: abnormality in cognitive function if MMSE <26; abnormality in locomotion if SPPB <10; abnormality in vitality if handgrip strength ≤Q1 (different values were applied for each sex); abnormality in psychological domain if GDS ≥5; Model 1: adjusted by sex, age (years), education (no diploma or primary school certificate, secondary education, high school diploma or university level) and their interaction with time. ***Model 2: adjusted by sex, age (years), education (no diploma or primary school certificate, secondary education, high school diploma or university level), BMI (kg/m^2^), number of comorbidities (considering diabetes, hypertension, hypercholesterolemia, cardiovascular disease, active cancer, asthma or chronic obstructive pulmonary disease) and their interaction with time.

In sensitivity analysis with IC status based on the presence of abnormalities in IC domains, significant declines in cognitive function were only observed among the group with low IC when MMSE was included in IC construction, with significant between-group mean differences in the unadjusted model (3 years: −0.17, 95 % CI −0.31 to −0.03; *p* = 0.018; 5 years: −0.21, 95 % CI −0.38 to −0.04; *p* = 0.016). Differences did not persist after adjustments for potential confounders though. According to the IC construct excluding the MMSE, significant declines in cognitive function were observed for participants with low IC both at 3 years (−0.15, 95 % CI −0.24 to −0.05; *p* = 0.002) and 5 years (−0.29, 95 % CI −0.41 to −0.17; *p* < 0.0001), but only at 5 years for those with normal IC (−0.13, 95 % CI −0.25 to −0.00; *p* = 0.044). However, no between-group mean differences were observed (Table 4).

## 4 Discussion

This study investigated cross-sectional and prospective associations between IC (defined by two different methods), plasma p-tau181 and cognitive function assessments among a sample of community-dwelling older adults at risk of cognitive decline. IC was associated with plasma p-tau181 and with the composite cognitive score (in the sense of higher IC as a protector of these cognitive outcomes), but findings varied according to method of IC classification and to the presence or absence of adjustments for potential confounders. In prospective analyses, we more specifically found a significant between-group change in p-tau181 levels over the 3-year follow in the fully adjusted model reflecting no change among the low IC group and a slight decline in the normal IC group.

Although crucial for understanding the global pathways of aging nowadays, the innovative concept of IC has no official nor consensual guideline for assessment so far [2,27]. Considering it encompasses several health domains, which can be, each of them, evaluated by multiple different scales and tests, a wide range of possibilities exists to build IC. In spite of the absence of data on the sensory domain of IC in our study clearly be a limitation, many other studies were also restricted to analyzing IC by only four out of its five domains, as recently identified by Sanchez-Rodriguez et al. [2] in a review of intervention studies targeting IC. Moreover, since there are no consensual cutoffs defined to establish low or normal IC, we have opted to use quartiles (for the composite IC score) and the presence of abnormalities in IC domains to classify participants according to their IC status. Given the importance of the concept of IC to clinical care and public health, it would be interesting if in the future, research could lean on standard IC assessments and validated cutoffs for investigating IC status.

To the best of our knowledge, no study has explored the associations between IC and plasma p-tau181 so far (nor other plasma or cerebrospinal fluid p-tau metabolites). In spite of the discrepancies in our findings depending on the type of analysis (cross-sectional or longitudinal; unadjusted or adjusted), our study shows initial evidence of a potential association between IC and this newer biomarker of AD. Further research exploring IC and plasma p-tau are therefore warranted. In the meanwhile, interesting recent evidence linking IC to adverse health outcomes related to neurodegeneration stimulates this search: in a cohort analysis including 366,406 participants from the UK Biobank, Sun et al. [16], have recently shown deficits in IC to be risk factors to the incidence of different types of dementia, including AD (hazard ratio [HR] = 2.17, 95 %CI 1.92 to 2.45). As expected, risk was markedly higher among subjects with IC deficits combined with genetic susceptibility to dementia (HR=8.11, 95 %CI 6.28 to 10.47) [16], highlighting the urgent need to promote IC protection over this subgroup.

Our study also explored the prospective associations between IC and cognitive function assessed by a composite cognitive score based on four tests. Although cognition is part of the IC construct, the clinical meaning of this concept proposed by the WHO goes beyond of just an agglomerate of health domains. Combined, they summarize individuals’ interrelated [15] composite of all the physical and mental capacities at any point in their lifetime [1], reflecting general health status. Anyway, in order to counteract this potential bias, we also performed the analyses by excluding the MMSE from the IC status. Results remained similar when using the 0–100 points IC score. For the classification based on abnormalities in IC domains, significant between-group differences in CCS change over time between low and normal IC were only observed in the unadjusted model with the MMSE kept in the IC variable, indicating more pronounced decline in cognitive function among those with low IC.

As neurodegeneration and cognitive decline typically occur slowly over time, several years before the diagnosis of MCI or dementia [28], and our sample was composed by individuals with high educational level (what indicates high cognitive reserve [29]), it is possible that more time would be necessary to more clearly observe the investigated associations. Another point to consider is the fact that findings might have been different if other plasma p-tau metabolites have been assessed. Ashton et al. [30] have shown that plasma p-tau217, but not p-tau231 nor p-tau181, was associated with disease progression in preclinical and prodromal AD among participants of the BioFINDER-1 cohort and the Wisconsin Registry for Alzheimer's Prevention cohort.

This is the first study to cross-sectionally and longitudinally investigate the relationship between IC and plasma p-tau181. As another strength of our study, we should mention the fact that IC was assessed by two different methods. On the other hand, some limitations should be noted. Plasma p-tau181 concentrations were analyzed in a subsample of participants from the randomized controlled MAPT trial. Moreover, assessment was performed in stored blood samples, some years after collection (in 2022). However, samples were adequately stored, and analyzed in the same laboratory (Clinical Neurochemistry Laboratory, University of Gothenburg, Mölndal, Sweden). As typically observed in longitudinal studies, some measures were missing for some subjects in the end of follow-up. In addition, as MAPT was not intentionally designed to assess IC, its database did not have information for the sensory domain of IC for most participants, so this domain was not used to build IC in the present study. Although MAPT was a trial involving omega-3 PUFA supplementation and multidomain intervention, only participants of the control group were included in the prospective analyses. Finally, considering that, generally, multidomain prevention trial participants are not representative of the general older population [31], comparing our findings with other populations demands caution.

## 5 Conclusions

The present study identified initial evidence of a potential association between IC and plasma p-tau181 among community-dwelling older adults. Lower IC was also associated with more pronounced cognitive decline after 3 years and 5 years in unadjusted analysis, according to an IC classification based on the presence of abnormalities on IC domains. Further research may help settle the role of IC as a predictor of neurodegenerative diseases such as AD. In this regard, interventions based on lifestyle changes and healthy habits (such as physical activity, healthy diets, stress management and cognitive training) have potential to protect IC over the aging process, to increase life quality and to prevent cognitive impairment and care dependency. Trials exploring multidomain interventions should be encouraged for a deeper comprehension on how to best promote IC protection (or even reverse IC declines) and reduce the burden of neurodegenerative diseases with clinical care and with public health policies.

## Funding

The MAPT study was supported by grants from the French Ministry of Health (PHRC 2008, 2009), University Hospital Center of Toulouse / Gérontopôle, Pierre Fabre Research Institute (manufacturer of the omega-3 supplement), Exonhit Therapeutics (biological sample collection) and Avid Radiopharmaceuticals (PET-amyloid measurement). The promotion of this study was supported by the University Hospital Center of Toulouse. The data sharing activity was supported by the Association Monegasque pour la Recherche sur la maladie d’Alzheimer (AMPA) and the UMR 1027 Unit INSERM-University of Toulouse III. HZ is a Wallenberg Scholar and a Distinguished Professor at the Swedish Research Council supported by grants from the Swedish Research Council (#2023-00356, #202201018 and #2019-02397), the European Union’s Horizon Europe research and innovation programme under grant agreement No 101053962, and Swedish State Support for Clinical Research (#ALFGBG-71320). No sponsor placed any restriction on this work or had any role in the design of the study, data collection, data analyses or interpretation, or in the preparation, review, or approval of the manuscript.

## Declaration of Generative AI and AI-assisted technologies in the writing process

No artificial intelligence (AI) was used in the writing process of this manuscript.

**MAPT/DSA Group**

MAPT Study Group

Principal investigator: Bruno Vellas (Toulouse); Coordination: Sophie Guyonnet; Project leader: Isabelle Carrié; CRA: Lauréane Brigitte; Investigators: Catherine Faisant, Françoise Lala, Julien Delrieu, Hélène Villars; Psychologists: Emeline Combrouze, Carole Badufle, Audrey Zueras; Methodology, statistical analysis and data management: Sandrine Andrieu, Christelle Cantet, Christophe Morin; Multidomain group: Gabor Abellan Van Kan, Charlotte Dupuy, Yves Rolland (physical and nutritional components), Céline Caillaud, Pierre-Jean Ousset (cognitive component), Françoise Lala (preventive consultation). The cognitive component was designed in collaboration with Sherry Willis from the University of Seattle, and Sylvie Belleville, Brigitte Gilbert and Francine Fontaine from the University of Montreal.

Co-Investigators in associated centres: Jean-François Dartigues, Isabelle Marcet, Fleur Delva, Alexandra Foubert, Sandrine Cerda (Bordeaux); Marie-Noëlle-Cuffi, Corinne Costes (Castres); Olivier Rouaud, Patrick Manckoundia, Valérie Quipourt, Sophie Marilier, Evelyne Franon (Dijon); Lawrence Bories, Marie-Laure Pader, Marie-France Basset, Bruno Lapoujade, Valérie Faure, Michael Li Yung Tong, Christine Malick-Loiseau, Evelyne Cazaban-Campistron (Foix); Françoise Desclaux, Colette Blatge (Lavaur); Thierry Dantoine, Cécile Laubarie-Mouret, Isabelle Saulnier, Jean-Pierre Clément, Marie-Agnès Picat, Laurence Bernard-Bourzeix, Stéphanie Willebois, Iléana Désormais, Noëlle Cardinaud (Limoges); Marc Bonnefoy, Pierre Livet, Pascale Rebaudet, Claire Gédéon, Catherine Burdet, Flavien Terracol (Lyon), Alain Pesce, Stéphanie Roth, Sylvie Chaillou, Sandrine Louchart (Monaco); Kristelle Sudres, Nicolas Lebrun, Nadège Barro-Belaygues (Montauban); Jacques Touchon, Karim Bennys, Audrey Gabelle, Aurélia Romano, Lynda Touati, Cécilia Marelli, Cécile Pays (Montpellier); Philippe Robert, Franck Le Duff, Claire Gervais, Sébastien Gonfrier (Nice); Yannick Gasnier and Serge Bordes, Danièle Begorre, Christian Carpuat, Khaled Khales, Jean-François Lefebvre, Samira Misbah El Idrissi, Pierre Skolil, Jean-Pierre Salles (Tarbes).

MRI group: Carole Dufouil (Bordeaux), Stéphane Lehéricy, Marie Chupin, Jean-François Mangin, Ali Bouhayia (Paris); Michèle Allard (Bordeaux); Frédéric Ricolfi (Dijon); Dominique Dubois (Foix); Marie Paule Bonceour Martel (Limoges); François Cotton (Lyon); Alain Bonafé (Montpellier); Stéphane Chanalet (Nice); Françoise Hugon (Tarbes); Fabrice Bonneville, Christophe Cognard, François Chollet (Toulouse).

PET scans group: Pierre Payoux, Thierry Voisin, Julien Delrieu, Sophie Peiffer, Anne Hitzel, (Toulouse); Michèle Allard (Bordeaux); Michel Zanca (Montpellier); Jacques Monteil (Limoges); Jacques Darcourt (Nice).

Medico-economics group: Laurent Molinier, Hélène Derumeaux, Nadège Costa (Toulouse).

Biological sample collection: Bertrand Perret, Claire Vinel, Sylvie Caspar-Bauguil (Toulouse).

Safety management: Pascale Olivier-Abbal

DSA Group: Sandrine Andrieu, Christelle Cantet, Nicola Coley.

## CRediT authorship contribution statement

**Kelly Virecoulon Giudici:** Writing – original draft. **Philipe de Souto Barreto:** Writing – review & editing. **Christelle Cantet:** Writing – review & editing, Formal analysis, Data curation. **Henrik Zetterberg:** Writing – review & editing, Methodology. **Kaj Blennow:** Writing – review & editing, Methodology. **Bruno Vellas:** Writing – review & editing, Project administration, Funding acquisition, Conceptualization.

## Declaration of competing interest

HZ has served at scientific advisory boards and/or as a consultant for Abbvie, Acumen, Alector, Alzinova, ALZpath, Amylyx, Annexon, Apellis, Artery Therapeutics, AZTherapies, Cognito Therapeutics, CogRx, Denali, Eisai, Enigma, LabCorp, Merry Life, Nervgen, Novo Nordisk, Optoceutics, Passage Bio, Pinteon Therapeutics, Prothena, Quanterix, Red Abbey Labs, reMYND, Roche, Samumed, Siemens Healthineers, Triplet Therapeutics, and Wave, has given lectures sponsored by Alzecure, BioArctic, Biogen, Cellectricon, Fujirebio, Lilly, Novo Nordisk, Roche, and WebMD, and is a co-founder of Brain Biomarker Solutions in Gothenburg AB (BBS), which is a part of the GU Ventures Incubator Program (outside submitted work). KB has served as a consultant and at advisory boards for Abbvie, AC Immune, ALZPath, AriBio, Beckman-Coulter, BioArctic, Biogen, Eisai, Lilly, Moleac Pte. Ltd, Neurimmune, Novartis, Ono Pharma, Prothena, Quanterix, Roche Diagnostics, Sanofi and Siemens Healthineers; has served at data monitoring committees for Julius Clinical and Novartis; has given lectures, produced educational materials and participated in educational programs for AC Immune, Biogen, Celdara Medical, Eisai and Roche Diagnostics; and is a co-founder of Brain Biomarker Solutions in Gothenburg AB (BBS), which is a part of the GU Ventures Incubator Program, outside the work presented in this paper. The other authors declare no conflicts.

## Appendix Supplementary materials

Image, application 1

Image, application 2
