# Supplementary material for: Associations between intrinsic capacity, plasma p-tau181 and cognitive function over a 5-year follow-up among community-dwelling older adults: a secondary analysis of the MAPT Study
Source: J Frailty Aging. 2025 Jul 1;14(4):100064. doi: 10.1016/j.tjfa.2025.100064 (PMC12399254; doi:10.1016/j.tjfa.2025.100064)
Supplement: Supplementary file 1 [file mmc1.docx]

**Supplementary Table 1.** Cross-sectional associations between baseline intrinsic capacity score divided in quartiles and plasma p-tau181 among community-dwelling older adults.

|  | |  | **Baseline plasma p-tau181** | | |
| --- | --- | --- | --- | --- | --- |
|  | |  | **β** | **95% CI** | **p-value** |
|  |  |  |  |  |  |
| Unadjusted | | Q1 | Ref. | Ref. | Ref. |
|  | | Q2 | -0.39 | -1.52 to 0.74 | 0.501 |
|  | | Q3 | -0.94 | -2.07 to 0.20 | 0.105 |
|  | | Q4 | -1.67 | -2.80 to -0.55 | **0.004** |
|  | |  |  |  |  |
| Adjusted (model 1) | | Q1 | Ref. | Ref. | Ref. |
|  | | Q2 | -0.40 | -1.51 to 0.71 | 0.477 |
|  | | Q3 | -0.50 | -1.64 to 0.64 | 0.391 |
|  | | Q4 | -1.06 | -2.21 to 0.09 | 0.072 |
|  | |  |  |  |  |
| Adjusted (model 2) | | Q1 | Ref. | Ref. | Ref. |
|  | | Q2 | -0.40 | -1.51 to 0.71 | 0.480 |
|  | | Q3 | -0.57 | -1.71 to 0.57 | 0.328 |
|  | | Q4 | -1.11 | -2.27 to 0.04 | 0.058 |

CI, confidence interval; IC, intrinsic capacity; Q1≤74.93; Q2 =74.94 to 79.57; Q3 = 79.58 to 83.56; Q4≥83.57; Model 1: adjusted by sex, age (years), education (no diploma or primary school certificate, secondary education, high school diploma or university level) and their interaction with time. Model 2: adjusted by sex, age (years), education (no diploma or primary school certificate, secondary education, high school diploma or university level), BMI (kg/m^2^), and number of comorbidities (considering diabetes, hypertension, hypercholesterolemia, cardiovascular disease, active cancer, asthma or chronic obstructive pulmonary disease).
