# Supplementary material for: Associations between intrinsic capacity, plasma p-tau181 and cognitive function over a 5-year follow-up among community-dwelling older adults: a secondary analysis of the MAPT Study
Source: J Frailty Aging. 2025 Jul 1;14(4):100064. doi: 10.1016/j.tjfa.2025.100064 (PMC12399254; doi:10.1016/j.tjfa.2025.100064)
Supplement: Supplementary file 2 [file mmc2.docx]

**Supplementary Table 2.** Mixed-effect linear regression analysis for variation in composite cognitive score over a 5-year follow-up according to baseline intrinsic capacity divided in quartiles among community-dwelling older adults (participants of the MAPT Study in the placebo group).

|  | |  |  | | **Change in composite cognitive score* from baseline to 3 or 5 years** | | | |  |  | **Group difference in composite cognitive score between IC quartiles over time** | | |
| --- | --- | --- | --- | --- | --- | --- | --- | --- | --- | --- | --- | --- | --- |
|  | |  |  | | **Within-group mean difference (95% CI)** | | | |  |  | **Between-group mean difference (95% CI); P-value** | | |
|  | |  |  | |  |  |  |  |  | **Unadjusted** | | **Adj. (model 1)** | **Adj. (model 2)** |
|  | **Intrinsic capacity score (0-100, MMSE included)** | | |  | |  |  |  |  |  | |  |  |
| Baseline to 3 years | |  |  | | Q1 | | -0.15 (-0.30, -0.01); **p=0.038** |  | Ref. |  | |  |  |
|  | |  |  | | Q2 | | -0.26 (-0.41, -0.12); **p=0.001** |  | Q2 vs. Q1 | -0.11 (-0.32, 0.10); p=0.290 | | -0.13 (-0.34, 0.08); p=0.217 | -0.12 (-0.33, 0.08); p=0.246 |
|  | |  |  | | Q3 | | -0.03 (-0.17, 0.11); p=0.703 |  | Q3 vs. Q1 | 0.13 (-0.08, 0.33); p=0.221 | | 0.02 (-0.19, 0.23); p=0.850 | 0.02 (-0.19, 0.23); p=0.833 |
|  | |  |  | | Q4 | | -0.02 (-0.16, 0.11); p=0.729 |  | Q4 vs. Q1 | 0.13 (-0.07, 0.33); p=0.205 | | -0.01 (-0.22, 0.21); p=0.929 | -0.01 (-0.22, 0.20); p=0.940 |
| Baseline to 5 years | |  |  | | Q1 | | -0.29 (-0.48, -0.11); **p=0.002** |  | Ref. |  | |  |  |
|  | |  |  | | Q2 | | -0.42 (-0.60, -0.24); **p<0.0001**  **38** |  | Q2 vs. Q1 | -0.13 (-0.39, 0.13); p=0.329 | | -0.11 (-0.36, 0.15); p=0.417 | -0.10 (-0.36, 0.15); p=0.431 |
|  | |  |  | | Q3 | | -0.07 (-0.25, 0.10); p=0.403 |  | Q3 vs. Q1 | 0.22 (-0.03, 0.47); p=0.087 | | 0.12 (-0.14, 0.37); p=0.370 | 0.13 (-0.12, 0.39); p=0.309 |
|  | |  |  | | Q4 | | -0.09 (-0.26, 0.07); p=0.249 |  | Q4 vs. Q1 | 0.20 (-0.05, 0.45); p=0.112 | | 0.06 (-0.20, 0.32); p=0.643 | 0.07 (-0.19, 0.33); p=0.595 |
|  | |  |  | |  | |  |  |  |  | |  |  |
|  | **Intrinsic capacity score (0-100, MMSE excluded)** | | |  | |  |  |  |  |  | |  |  |
| Baseline to 3 years | |  |  | | Q1 | | -0.16 (-0.31, -0.01); **p=0.034** |  | Ref. |  | |  |  |
|  | |  |  | | Q2 | | -0.18 (-0.32, -0.04); **p=0.014** |  | Q2 vs. Q1 | -0.02 (-0.23, 0.19); p=0.853 | | -0.04 (-0.24, 0.17); p=0.706 | -0.03 (-0.23, 0.17); p=0.765 |
|  | |  |  | | Q3 | | -0.13 (-0.27, 0.02); p=0.083 |  | Q3 vs. Q1 | 0.04 (-0.17, 0.24); p=0.736 | | -0.07 (-0.28, 0.14); p=0.506 | -0.07 (-0.28, 0.14); p=0.519 |
|  | |  |  | | Q4 | | 0.00 (-0.14, 0.14); p=0.987 |  | Q4 vs. Q1 | 0.16 (-0.04, 0.36); p=0.122 | | 0.00 (-0.22, 0.22); p=0.986 | 0.00 (-0.21, 0.22); p=0.982 |
| Baseline to 5 years | |  |  | | Q1 | | -0.31 (-0.50, -0.12); **p=0.002** |  | Ref. |  | |  |  |
|  | |  |  | | Q2 | | -0.33 (-0.50, -0.15); **p=0.0003**  **38** |  | Q2 vs. Q1 | -0.02 (-0.28, 0.24); p=0.869 | | -0.03 (-0.28, 0.22); p=0.833 | -0.02 (-0.27, 0.24); p=0.895 |
|  | |  |  | | Q3 | | -0.19 (-0.37, -0.02); **p=0.032** |  | Q3 vs. Q1 | 0.11 (-0.14, 0.37); p=0.388 | | 0.03 (-0.22, 0.29); p=0.790 | 0.04 (-0.22, 0.30); p=0.773 |
|  | |  |  | | Q4 | | -0.06 (-0.23, 0.10); p=0.457 |  | Q4 vs. Q1 | 0.24 (-0.01, 0.49); p=0.056 | | 0.08 (-0.19, 0.34); p=0.567 | 0.09 (-0.17, 0.36); p=0.490 |

CI, confidence interval; IC, intrinsic capacity; MMSE, Mini Mental State Examination; Q, quartile; *based on the sum of Z-scores from four cognitive tests divided by 4: free and total recall of the Free and Cued Selective Reminding test, the ten orientation items of the Mini-Mental State Examination, Digit Symbol Substitution Test and Category Naming Test; Model 1: adjusted by sex, age (years), education (no diploma or primary school certificate, secondary education, high school diploma or university level) and their interaction with time. Model 2: adjusted by sex, age (years), education (no diploma or primary school certificate, secondary education, high school diploma or university level), BMI (kg/m^2^), number of comorbidities (considering diabetes, hypertension, hypercholesterolemia, cardiovascular disease, active cancer, asthma or chronic obstructive pulmonary disease) and their interaction with time.
